# Supplementary material for: A yeast phenomic model for the influence of Warburg metabolism on genetic buffering of doxorubicin
Source: Cancer Metab. 2019 Oct 23;7:9. doi: 10.1186/s40170-019-0201-3 (PMC6806529; doi:10.1186/s40170-019-0201-3)
Supplement: Supplementary file 9 — Additional file 9. GO term-specific heatmaps for REMc/GTF-enriched clusters. GO term-specific heatmaps for significant GO process terms were generated as described in methods and Figs. 3 and 4. Any related child terms are presented in subsequent pages of the parent file name. GO terms with more than 100 children, with 2 or fewer genes annotated to the term, or a file size over 300KB are not shown. All heatmaps are generated with the same layout (see Figs. 3 and 4). [file 40170_2019_201_MOESM9_ESM.bz2 › Additional_File9_GOTermHeatmaps/Additional_File9_GOTermHeatmap/protein_folding_in_endoplasmic_reticulum.pdf]

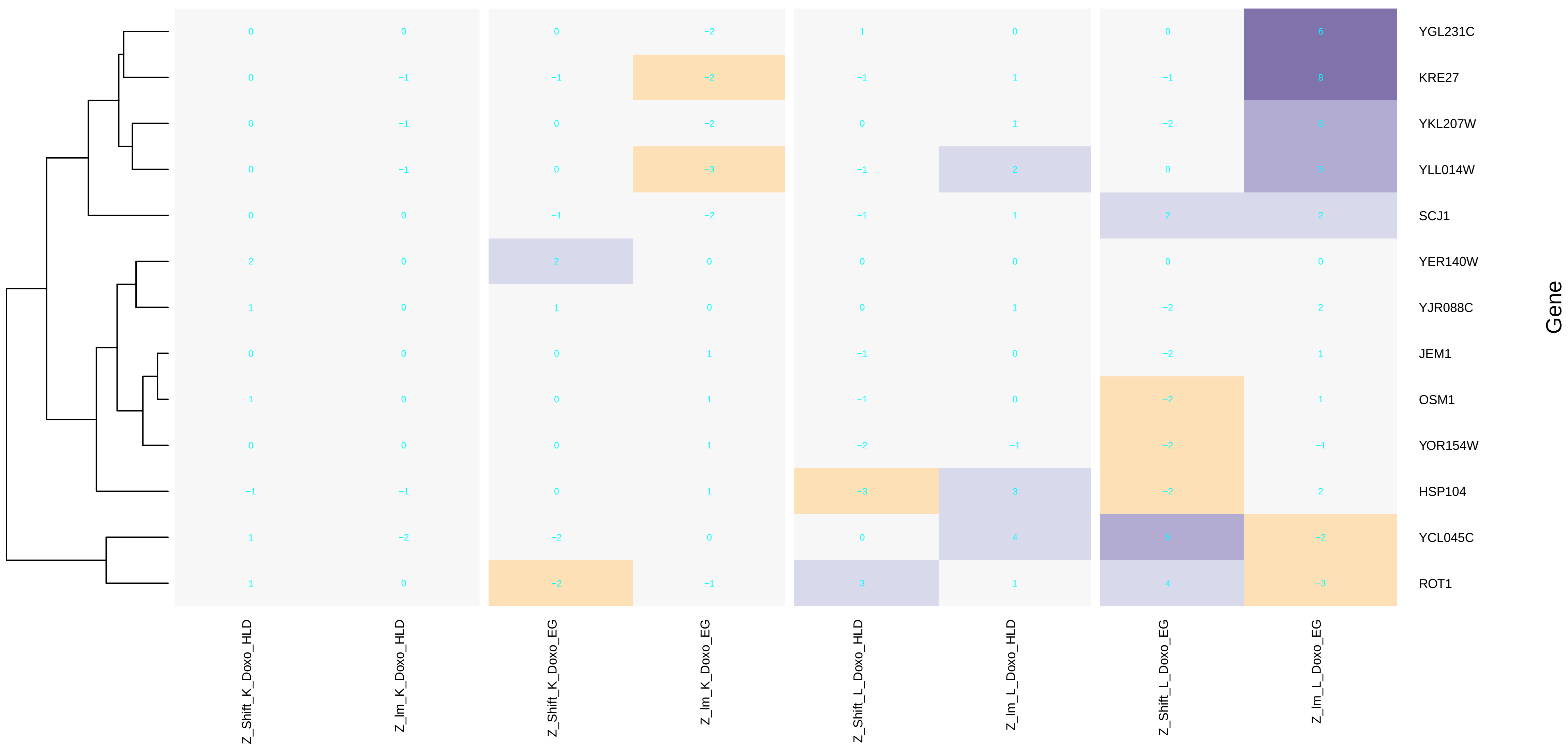

YGL231C 0 0 0 −2 1 0 0 6
KRE27 0 −1 −1 −2 −1 1 −1 8
YKL207W 0 −1 0 −2 0 1 −2 6
YLL014W 0 −1 0 −3 −1 2 0 5
SCJ1 0 0 −1 −2 −1 1 2 2
YER140W 2 0 2 0 0 0 0 0
YJR088C 1 0 1 0 0 1 −2 2
JEM1 0 0 0 1 −1 0 −2 1
OSM1 1 0 0 1 −1 0 −2 1
YOR154W 0 0 0 1 −2 −1 −2 −1
HSP104 −1 −1 0 1 −3 3 −2 2
YCL045C 1 −2 −2 0 0 4 5 −2
ROT1 1 0 −2 −1 3 1 4 −3
Z_Shift_K_Doxo_HLD
Z_Im_K_Doxo_HLD
Z_Shift_K_Doxo_EG
Z_Im_K_Doxo_EG
Z_Shift_L_Doxo_HLD
Z_Im_L_Doxo_HLD
Z_Shift_L_Doxo_EG
Z_Im_L_Doxo_EG
Gene
